# Whole exome sequencing identifies a new mutation in the *SLC19A2* gene leading to thiamine‐responsive megaloblastic anemia in an Egyptian family

**DOI:** 10.1002/mgg3.777

**Published:** 2019-05-29

**Authors:** Khalda Amr, Patrycja Pawlikowska, Said Aoufouchi, Filippo Rosselli, Ghada El‐Kamah

**Affiliations:** ^1^ Human Genetics and Genome Research Division, National Research Centre Cairo Egypt; ^2^ UMR8200‐CNRS, Gustave Roussy Villejuif Cedex France; ^3^ Université Paris‐Sud, Université Paris‐Saclay Orsay France; ^4^ Equipe labellisée "La Ligue Contre le Cancer" Villejuif Cedex France

**Keywords:** bone marrow failure, deafness, diabetes, *SLC19A2*, thiamine‐responsive megaloblastic anemia, THTR‐1

## Abstract

**Background:**

The Solute Carrier Family 19 Member 2 (*SLC19A2*, OMIM *603941) encodes the thiamine transporter 1 (THTR‐1) that brings thiamine (Vitamin B1) into cells. THTR‐1 is the only thiamine transporter expressed in bone marrow, cochlear, and pancreatic beta cells. THTR‐1 loss‐of‐function leads to the rare recessive genetic disease Thiamine‐Responsive Megaloblastic Anemia (TRMA, OMIM #249270).

**Methods:**

In vitro stimulated blood lymphocytes were used for cytogenetics and the isolation of genomic DNA used to perform whole exome sequencing (WES). To validate identified mutations, direct Sanger sequencing was performed following PCR amplification.

**Results:**

A 6‐year‐old male born from a consanguineous couple presenting bone marrow failure and microcephaly was referred to our clinic for disease diagnosis. The patient presented a normal karyotype and no chromosomal fragility in response to DNA damage. WES analysis led to the identification of a new pathogenic variant in the *SLC19A2* gene (c.596C>G, pSer199Ter) allowing to identify the young boy as a TRMA patient.

**Conclusion:**

Our analysis extend the number of inactivating mutations in *SLC19A2* leading to TRMA that could guide future prenatal diagnosis for the family and follow‐up for patients.

## 1 INTRODUCTION

The association of megaloblastic anemia, diabetes mellitus, and sensorineural deafness is recognized as a rare recessive genetic disease named Thiamine‐Responsive Megaloblastic Anemia (TRMA, OMIM #249270) also called thiamine metabolism dysfunction syndrome‐1 (THMD1) or Roger's syndrome (Porter, Rogers, & Sidbury, 1969). The responsible gene, *SLC19A2* (OMIM: *603941, HGNC ID: 10938; NM_006996.2) is composed of six exons, spanning 22.5 kb on the chromosome 1q and codes for the high‐affinity thiamine transporter protein THTR‐1 and was identified in 1999 (Diaz, Banikazemi, Oishi, Desnick, & Gelb, 1999; Fleming et al., 1999; Labay et al., 1999). Long of 497aa, the encoded *SLC19A2* protein*,* THTR‐1 (Uniprot/Swissprot: O60779) possesses 12 putative transmembrane domains and is the sole thiamine transporter expressed in bone marrow, in a subset of cochlear cells and in pancreatic beta cells, explaining the clinical triad that defines TRMA (Diaz et al., 1999; Fleming et al., 1999; Labay et al., 1999). However, TRMA patients could also present several other clinical abnormalities, including thrombocytopenia, pancytopenia, myelodysplasia, cone‐rod dystrophy, optic atrophy, retinal degeneration, ataxia, hepatomegaly, atrial septal defect, and stroke that make difficult diagnosis and genotype phenotype correlation analysis (Shaw‐Smith et al., 2012). This variability could be associated with differences in residual activities of some mutants, to environmental clues and/or coinheritance with mutations in other genes. Indeed, less than 100 families associated with around 60 *SLC19A2* variants have been described.

## 2 METHODS

### 2.1 Ethical compliance

This study obtained the approbation of a genetic study by the Ethics Committee of the National Research Center of Cairo and the written informed consent of the parents.

### 2.2 Lymphocytes culture and DNA analysis

Blood samples were obtained from patient and parents and lymphocytes cultures were established following standard protocol and used for cytogenetics analysis. Whole exome sequencing (WES) on genomic DNA isolated from blood samples were performed following a standard protocol. Direct Sanger sequencing of the *SLC19A2* gene(OMIM: *603941, HGNC ID: 10938; NM_006996.2) was performed using standard methods following PCR amplification with the following primers: *SLC19A2* ‐ Rev 5′‐AGGCTGAGCTTACCGGTTC‐3′; *SLC19A2* ‐ 5′‐CCAGTATGGACTTACTCTTACCTGG‐3′.

## 3 RESULTS

**Figure 1:**
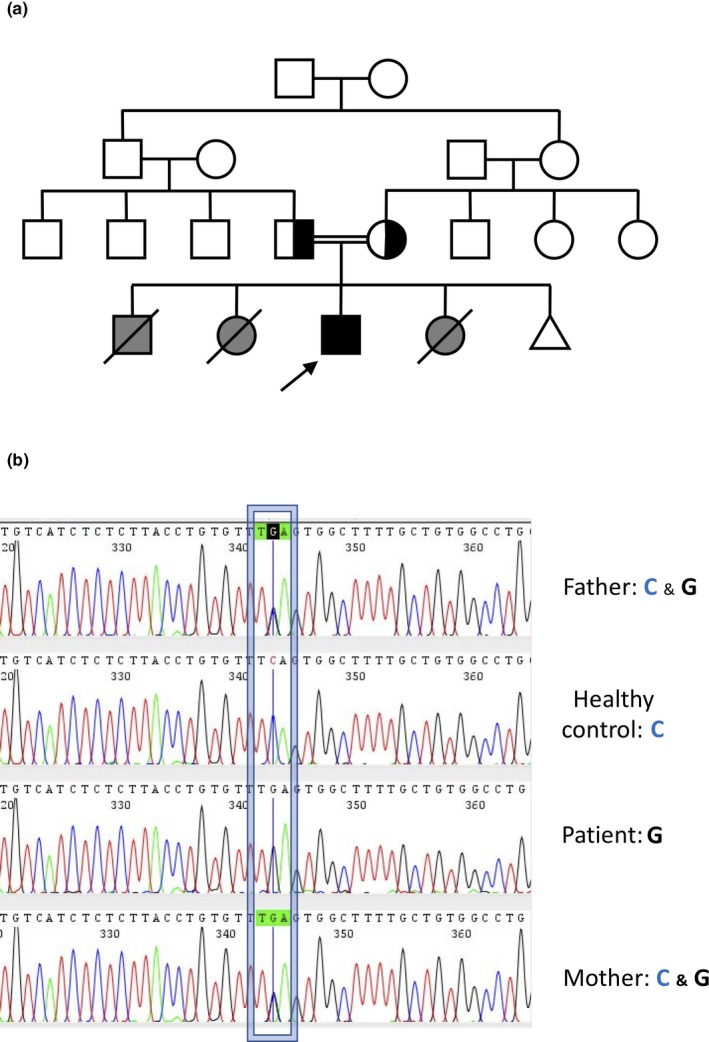
(a) pedigree of the index patient showing a typical autosomal recessive pattern of inheritance. Since siblings were already died at the time of our proband analysis, TRMA is proposed only on the basis of their clinical characteristics. (b) Sequences showing the novel point mutation identified in the exon 2 of *SLC19A2* (OMIM: *603941, HGNC ID: 10938; NM_006996.2)

A 6‐year‐old boy presenting with bone marrow failure (BMF) and microcephaly (−3,6), hearing and language impairment, as well as intellectual disability (SQ 57 by Vineland social maturity scale) was admitted at the Hereditary Blood Disorders Clinic, Human Genetics and Genome Research Division of the National Research Centre, Cairo, Egypt. The patient is born in a consanguineous family, marriage between first cousins, and was preceded by two infants and followed by one. The first sibling died at the age of 6 months from severe ear infection, the second had diabetes and BMF and died at age of 2 years, the last sib, died at the age of 9 months, was also diagnosed as diabetic at the age of 7 months and presented an anemic status. A fifth pregnancy did not come to its end (Figure 1a).

Initial suspicion of Fanconi anemia, the most frequent inherited bone marrow failure syndrome associated with mutations in more than 20 genes (Gueiderikh, Rosselli, & Neto, 2017), was rejected on the basis of the analysis of the chromosomal sensitivity to the exposure to DNA interstrands crosslinking inducing agent Dyepoxybutane (data not shown).

Considering the consanguinity of the family and the health problems of the siblings, a disorder inherited as an autosomal recessive trait was suspected. Thus, taking into account the variability of the phenotype and the little information on the metabolism of the patient we have, we chosen a holistic approach rather than a gene panel or a candidate gene approach to identify the gene‐coding protein which loss‐of‐function was responsible for the disease. Whole exome sequencing (WES) was performed on genomic DNA isolated from blood samples obtained from the parents and the patient. After filtering to exclude known nonpathogenic SNPs, eliminating "silent" variations (synonymous SNV, nonframeshift insertion, nonframeshift deletion) and SNPs present in the 1000G and AvSNP150 data bases, around 15,000 variants were identified in each individual. Among the variants presenting an allelic frequency (VAF) >25% and <75% in the parents, only two, the c. 596C>G in the exon 2 of the *SLC19A2* gene and the c.3438T>G in the exon 26 of the *VWF* gene, were scored as «likely pathogenics». Both genomic variants lead to a stop codon in the mRNA. Of the two variants, only the *SLC19A2* c.596C>G presented a VAF of 100, that is homozygosity, in the patient. Mutation was validated by Sanger analysis (Figure 1b). The identified C to G transversion leads to a codon stop that truncates the THTR‐1 receptor at Ser199 (Ser199Ter) in the middle of the sixth transmembrane domain of the protein.

## 4 DISCUSSION

Taken into account the clinical features and the identified mutated gene, we conclude that the patient is affected by TRMA. The identified mutation was not previously described. The identification of the inactivating mutation in the family will allow prenatal diagnosis for future pregnancy in the family and for follow‐up and treatment of eventual new siblings. Indeed, lifelong oral treatment with thiamine at pharmacological doses (25‐100 mg/day) reverse anemia and delay diabetes, and, when started during the first years of life, prevent hearing defects.

Our patient is the second Egyptian with a pathogenic mutation in the *SLC19A2* gene. The first one was identified by direct sequencing of known genes involved in diabetes (Habeb et al., 2018). As for our proband, the first Egyptian patient identified in the study of Habeb and collaborators was from consanguineous parents. He/she presented in addition to diabetes, deafness, anemia, thrombocytopenia, mild leukopenia, and atrial septal defect. He/she presented a previously unknown homozygous mutation in the fourth exon of the *SLC19A2* gene that leads to a stop codon (p.Trp387Ter) resulting in a protein truncated at the beginning of the ninth transmembrane domain.

Thus, it seems that *SLC19A2* inactivating mutations are diffused in the Egyptian genetic background and are different from those already retrieved worldwide. On the previous basis it appears interesting to propose the inclusion of the *SLC19A2 gene* in the panels of candidate genes used for the diagnosis of complex diseases associating bone marrow failure, diabetes and hearing loss and, possibly, developmental anomalies of heart and bones (microcephaly).

## DISCLOSURE

The authors have no conflict of interest to declare.
